# Delayed Renal Artery Stump Pseudoaneurysm and Arteriovenous Fistula Following Nephrectomy Complicated by Pulmonary Coil Migration: Successful Laparoscopic Management

**DOI:** 10.3390/jcm15218555

**Published:** 2026-11-03

**Authors:** Paulina Mizera, Karol Kamil Kłosiński, Joanna Maria Czyżewska, Jacek Cywiński, Zbigniew Włodzimierz Pasieka, Mateusz Jęckowski

**Affiliations:** 1 Department of Vascular Surgery, The Nicolaus Copernicus Provincial Multispecialty Center for Oncology and Traumatology in Lodz, Pabianicka 62, 93-513 Lodz, Poland; 2 Department of Biomedicine and Experimental Surgery, Medical University of Lodz, Narutowicza 60, 90-136 Lodz, Polandzbigniew.pasieka@umed.lodz.pl (Z.W.P.); 3 Department of General and Oncological Surgery, Maria Skłodowska-Curie Provincial Specialist Hospital in Zgierz, Parzęczewska 35, 95-100 Zgierz, Poland; 4 Department of Surgical Oncology, The Nicolaus Copernicus Provincial Multispecialty Center for Oncology and Traumatology in Lodz, Pabianicka 62, 93-513 Lodz, Poland

**Keywords:** renal arteriovenous fistula, renal artery stump pseudoaneurysm, nephrectomy, coil migration, endovascular embolization, laparoscopic ligation

## Abstract

**Background:** Renal arteriovenous fistula (RAVF) and renal artery pseudoaneurysm (RAP) are rare vascular complications that may occur following renal surgery, trauma, or endovascular interventions. Delayed presentation after nephrectomy is exceptionally uncommon. **Case Description:** We report the case of a 59-year-old woman with a pseudoaneurysm of the left renal artery stump associated with a high-flow renal arteriovenous fistula who was diagnosed three decades after left nephrectomy performed for complicated nephrolithiasis. The patient presented with episodic left flank pain without haematuria. Computed tomography angiography (CTA) and selective angiography confirmed the presence of a pseudoaneurysm and a high-flow arteriovenous fistula. Endovascular coil embolization was initially attempted; however, the coil migrated through the high-flow fistulous tract into the peripheral left pulmonary arterial circulation. Because the patient remained asymptomatic and haemodynamically stable and the coil was located peripherally, the procedural risk of retrieval was considered to outweigh the anticipated benefit. The coil was therefore left in situ under clinical and imaging surveillance. Because substantial arteriovenous shunting persisted after the unsuccessful embolization, definitive laparoscopic ligation of the left renal artery and vein stumps was successfully performed approximately one month later. During the two-year follow-up, two CTA examinations and Doppler ultrasonography confirmed complete occlusion of the fistula and pseudoaneurysm, with no recurrent abdominal symptoms or pulmonary complications related to the migrated coil. **Discussion:** Although post-nephrectomy arteriovenous fistulas presenting 28 and 45 years after surgery have previously been reported, the combination of a renal artery stump pseudoaneurysm, a high-flow fistula, pulmonary coil migration, persistent arteriovenous shunting, and subsequent successful laparoscopic management is particularly unusual. This case highlights the technical challenges of endovascular treatment in high-flow fistulas and the role of surgical management following unsuccessful embolization. **Conclusions:** Delayed renal artery stump pseudoaneurysm associated with renal arteriovenous fistula after nephrectomy is an extremely rare condition. Although endovascular embolization remains the preferred first-line treatment, surgical management may be necessary in cases complicated by technical failure or embolization-related complications.

## 1. Introduction

Renal arteriovenous fistulas (RAVFs) and renal artery pseudoaneurysms (RAPs) are uncommon vascular lesions associated with renal interventions, trauma, and surgery. Acquired fistulas may present with haematuria, flank pain, hypertension, or high-output cardiac failure, although symptoms can be subtle. Duplex ultrasonography can demonstrate abnormal shunt flow, while computed tomography angiography (CTA) defines the anatomy and catheter angiography permits detailed assessment and potential treatment [1,2,3,4,5].

Delayed vascular lesions arising from the renal pedicle after nephrectomy are particularly unusual. Historical reports have documented post-nephrectomy fistulas presenting 28 and 45 years after surgery [6,7]. Treatment has included open surgical closure and endovascular vascular plugs. However, the optimal rescue strategy when embolization of a high-flow post-nephrectomy fistula fails, particularly after embolic material reaches the pulmonary circulation, remains poorly described.

Recent reports have described transvenous retrieval of migrated coils with intraprocedural pulmonary circulation protection [8], Fogarty-assisted embolization [9], simultaneous transarterial and transvenous balloon-assisted coil embolization [10], and balloon-assisted coil embolization with flow control in high-flow RAVFs [11]. These approaches emphasize the importance of flow control and embolic-device stability. We report a renal artery stump pseudoaneurysm associated with a high-flow arteriovenous fistula presenting approximately 30 years after nephrectomy, complicated by pulmonary coil migration during attempted embolization and subsequently treated by laparoscopic vascular stump closure. The report focuses on the rationale for rescue surgery, technical considerations, and the limits of conservative management of migrated embolic material.

## 2. Case Description

A 59-year-old woman was electively admitted to the Department of Vascular Surgery with a diagnosis of a symptomatic pseudoaneurysm of the left renal artery stump. Her medical history included a left nephrectomy performed three decades earlier for complicated nephrolithiasis with hydronephrosis.

The patient reported episodic left flank pain exacerbated by stress and physical activity but denied symptoms suggestive of recurrent urolithiasis. Her past medical history was significant for arterial hypertension, bronchial asthma, and varicose veins. She had no history of infectious diseases or drug allergies, and her family history was unremarkable. Her regular medications included esomeprazole 20 mg daily, ramipril 10 mg daily, amlodipine 10 mg daily, atorvastatin 20 mg daily, and inhaled beclomethasone 100 µg/formoterol 6 µg twice daily.

**Figure 1:**
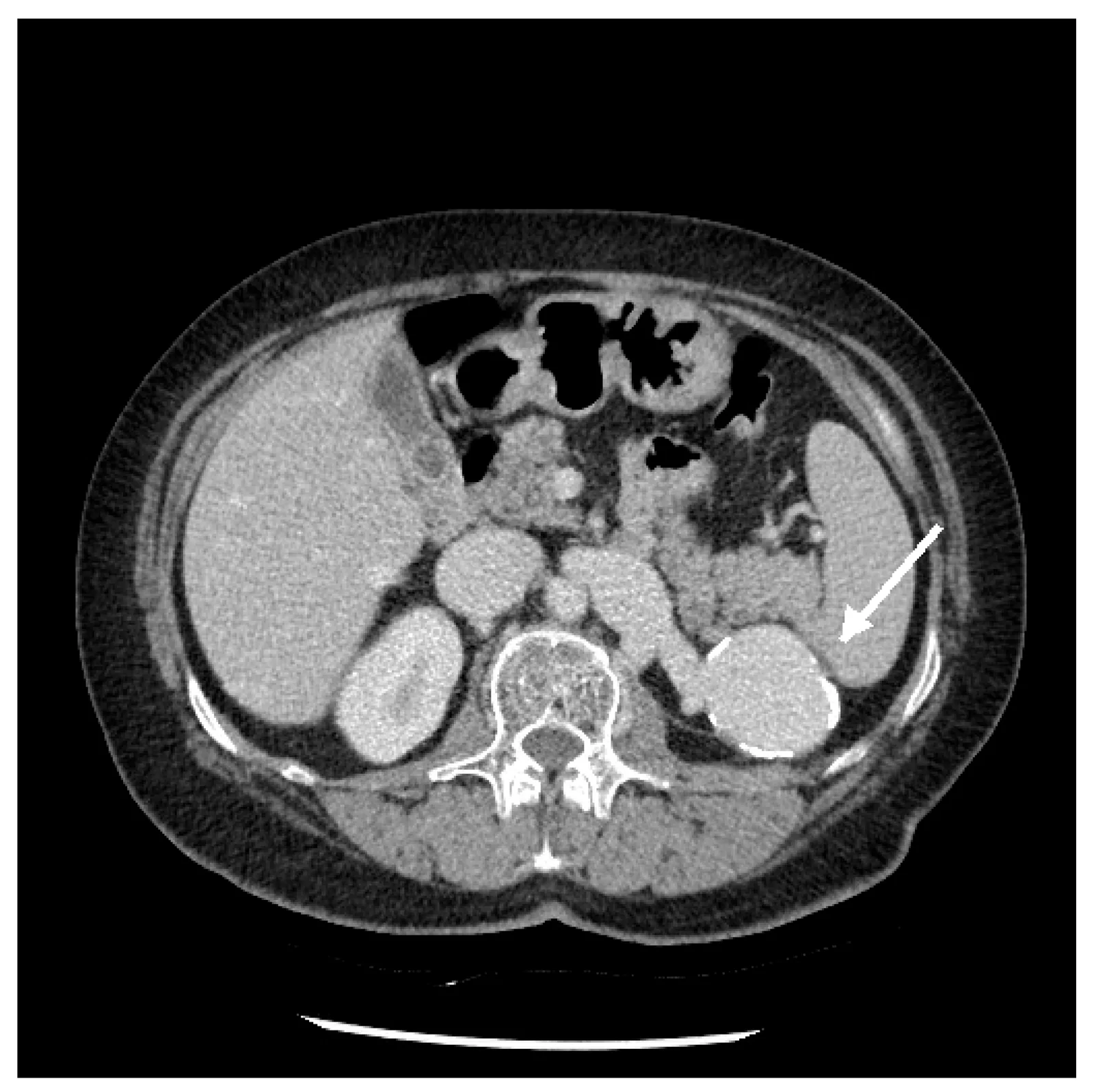
Contrast-enhanced CTA demonstrating a pseudoaneurysm arising from the left renal artery stump (53 × 43 × 41 mm). The white arrow indicates the pseudoaneurysm.

**Figure 2:**
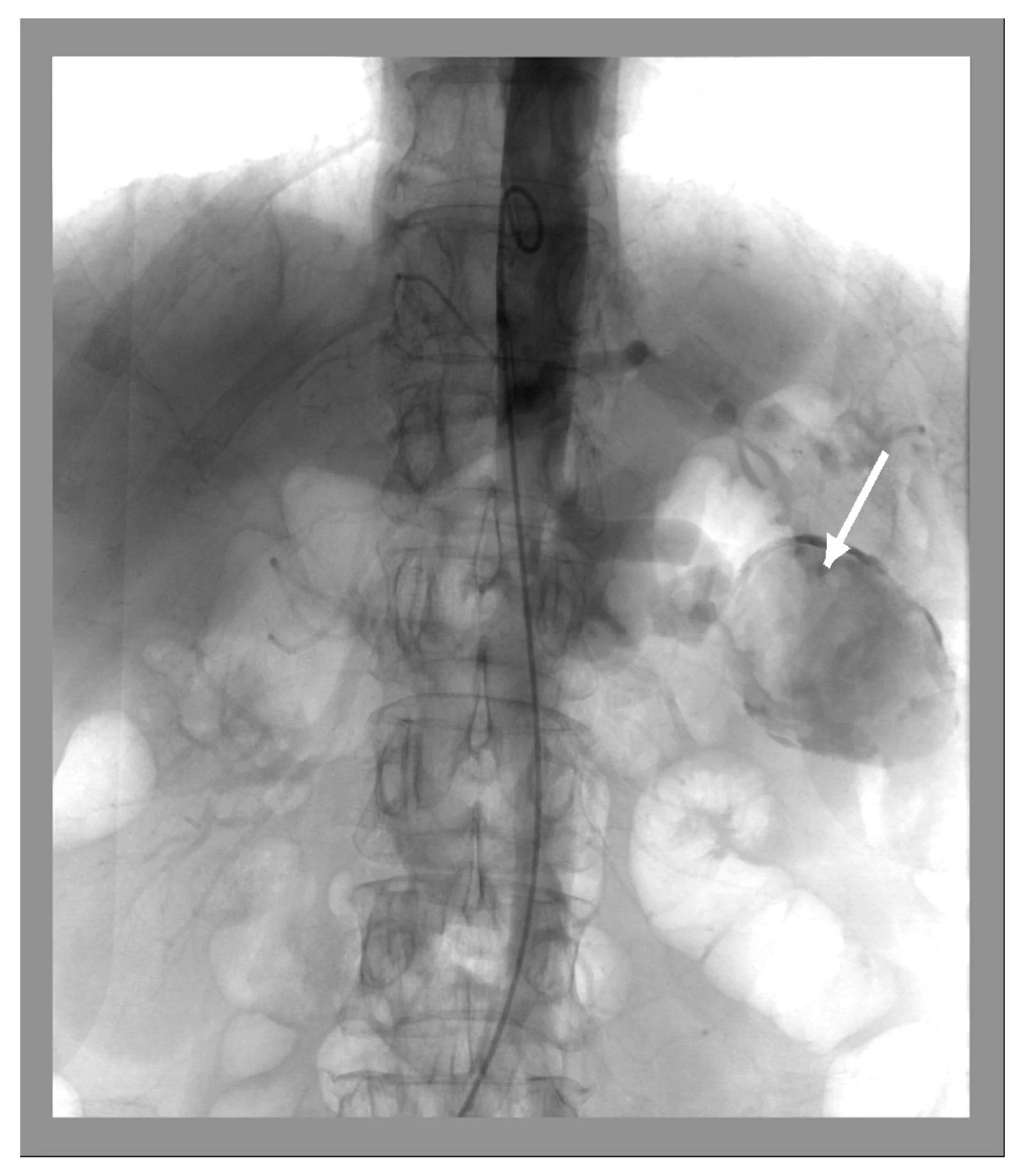
Selective angiography demonstrating the renal artery stump pseudoaneurysm with an associated high-flow arteriovenous fistula. The white arrow indicates the pseudoaneurysm.

On admission, laboratory test results were within normal limits. The patient had been referred to our department with a previously performed CTA demonstrating a pseudoaneurysm of the left renal artery stump measuring 53 × 43 × 41 mm (Figure 1). No preliminary Doppler ultrasonography was performed before CTA. Selective angiography was subsequently performed for detailed anatomical assessment and treatment planning and confirmed a high-flow arteriovenous fistula between the left renal artery stump and the left renal vein (Figure 2). The fistulous communication was identified before coil deployment.

**Figure 3:**
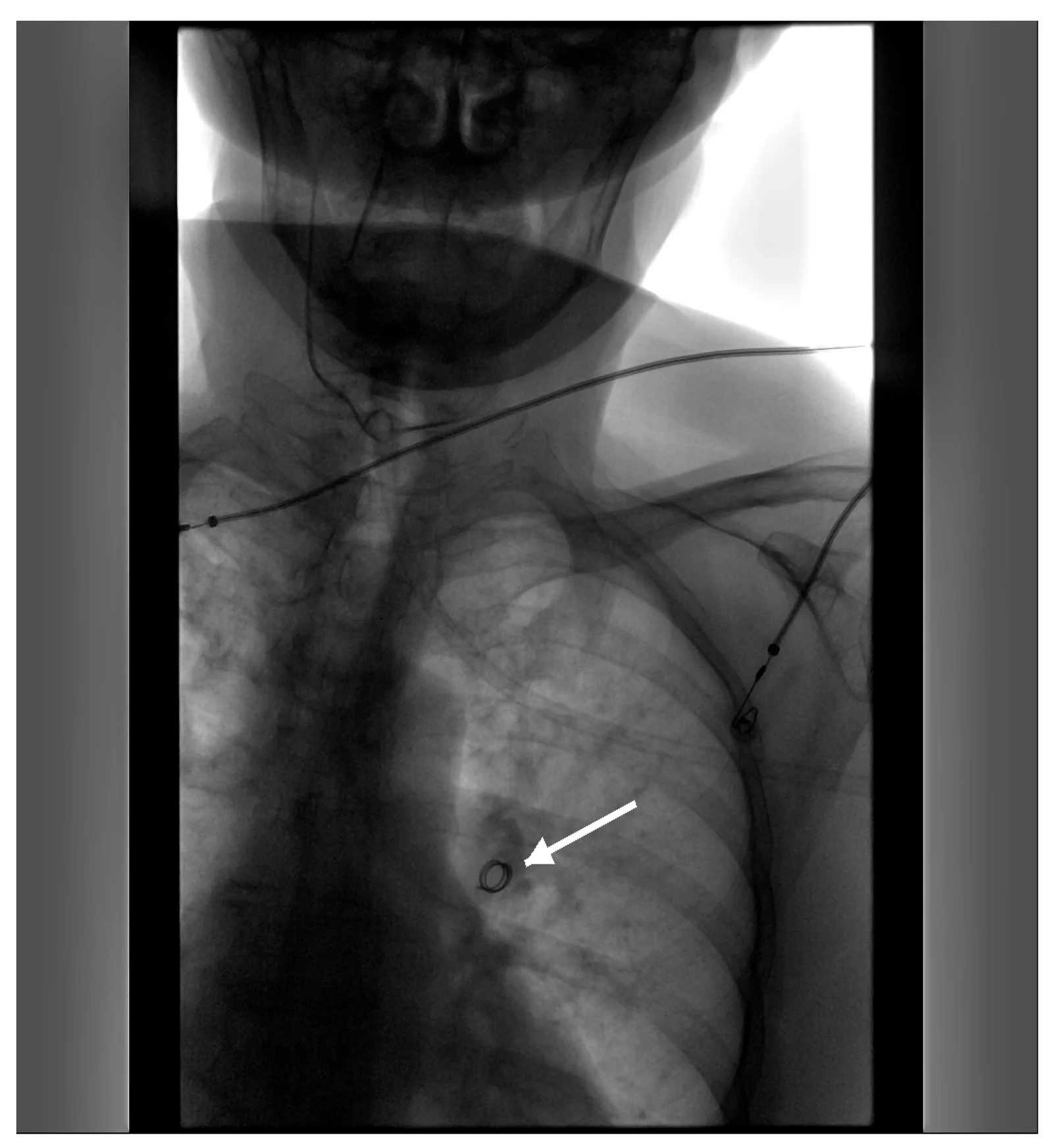
Embolization coil visualized in the left pulmonary arterial system following migration. The white arrow indicates the migrated coil.

Endovascular embolization of the left renal artery stump was attempted using an MReye^®^ embolization coil (IMMWCE-35-8-8; Cook Medical, Bloomington, IN, USA). During the procedure, migration of the embolization coil occurred through the fistulous communication into the left renal vein and subsequently into the left pulmonary arterial circulation. The patient remained haemodynamically stable and did not develop chest pain, haemoptysis, or dyspnoea. Follow-up imaging confirmed coil migration into the left pulmonary arterial system without evidence of pulmonary infarction or haemodynamic compromise (Figure 3).

Following migration, an intraoperative medical consultation was held to assess the risks and benefits of coil retrieval. Given the peripheral location of the coil within the pulmonary arterial circulation, the absence of respiratory symptoms, and the patient’s haemodynamic stability, the team judged that the risks of retrieval outweighed its anticipated benefits. The coil was therefore left in situ, with a plan for clinical and imaging surveillance.

**Figure 4:**
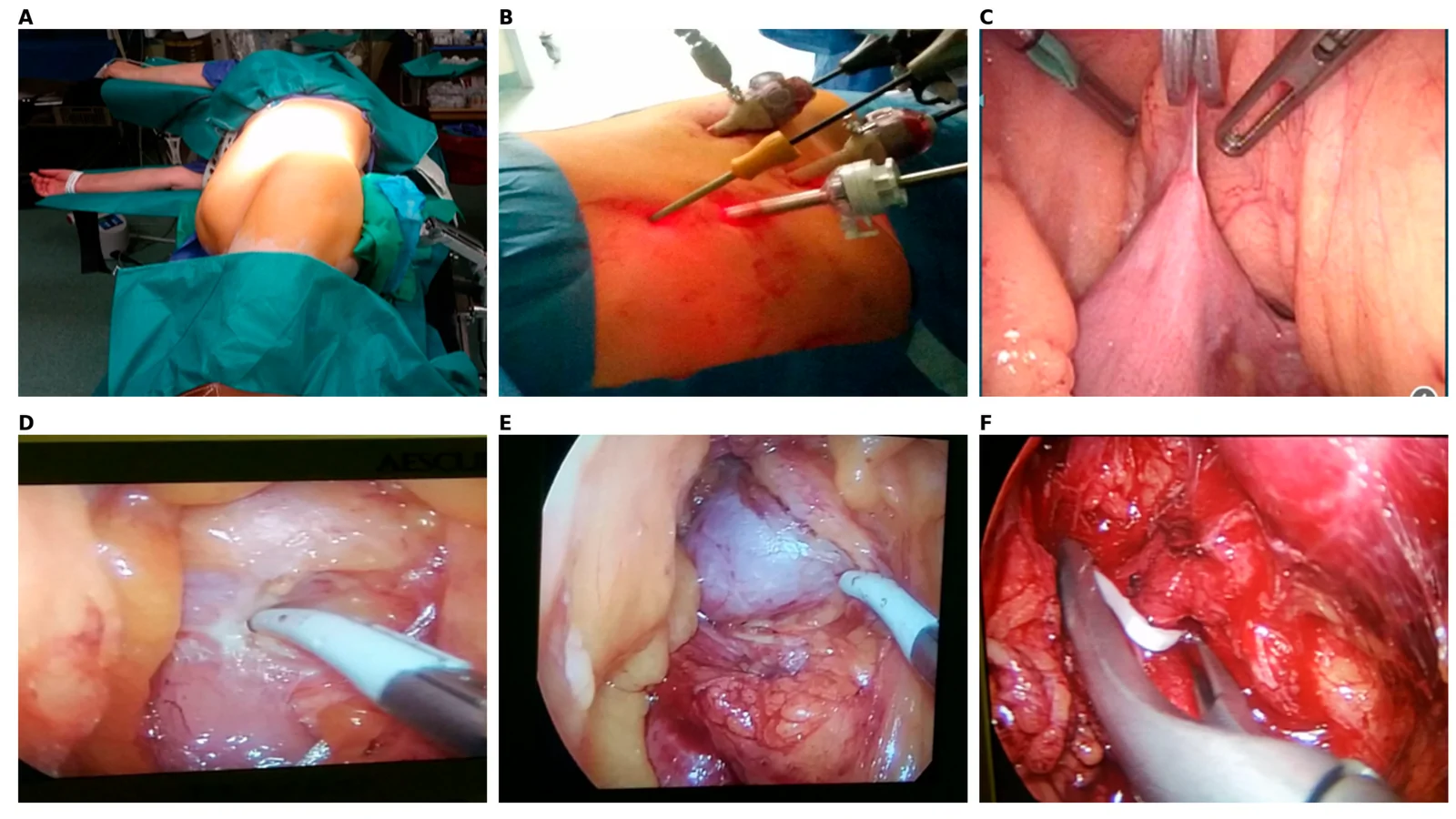
Laparoscopic approach and ligation of the left renal vascular stump. (**A**) Patient positioning; (**B**) laparoscopic trocar placement; (**C**) identification of the ligament of Treitz; (**D**) exposure of the left renal vascular stump; (**E**) identification of the dilated left renal vein; (**F**) stapling and occlusion of the vascular stump.

Because significant arteriovenous flow persisted despite the attempted embolization, definitive surgical treatment was undertaken. Laparoscopic ligation of the left renal vascular stump was performed under general anesthesia (Figure 4).

Preoperative computed tomography angiography was used to assess the vascular anatomy and demonstrated relatively long renal vascular stumps. This finding was considered favourable for laparoscopic dissection and secure vascular stapling and was used to inform the decision not to establish prophylactic arterial access.

Given the risk of pseudoaneurysm rupture and major haemorrhage, a contingency plan for emergency vascular control was established. Laparoscopic Satinsky clamps were prepared for potential aortic cross-clamping. A dedicated hybrid operating room was not available at the time of surgery; however, the femoral arterial access site was prepared and draped, and a mobile C-arm was available for emergency angiography and potential intra-arterial balloon occlusion. No arterial cannula or sheath was inserted prophylactically because the favourable vascular anatomy did not justify the additional access-related risk. If uncontrolled bleeding occurred, urgent arterial cannulation and balloon occlusion were planned to reduce blood loss during conversion to open surgery. These rescue measures were not required.

Intraoperatively, only mild intra-abdominal adhesions were encountered. The ligament of Treitz was identified, and the operative field was developed to expose the abdominal aorta and the left renal vascular pedicle. The left renal vein was identified and was markedly dilated, consistent with chronic high-flow arteriovenous shunting.

After careful dissection and adequate exposure of the renal vascular pedicle, the left renal vein was closed using an endoscopic vascular stapler (Endo GIA™, 2.5 mm; Medtronic, Minneapolis, MN, USA), while the proximal left renal artery was secured with Hem-o-lok^®^ clips (Teleflex Medical, Morrisville, NC, USA). This achieved complete interruption of flow through the fistulous communication. No intraoperative complications occurred.

**Figure 5:**
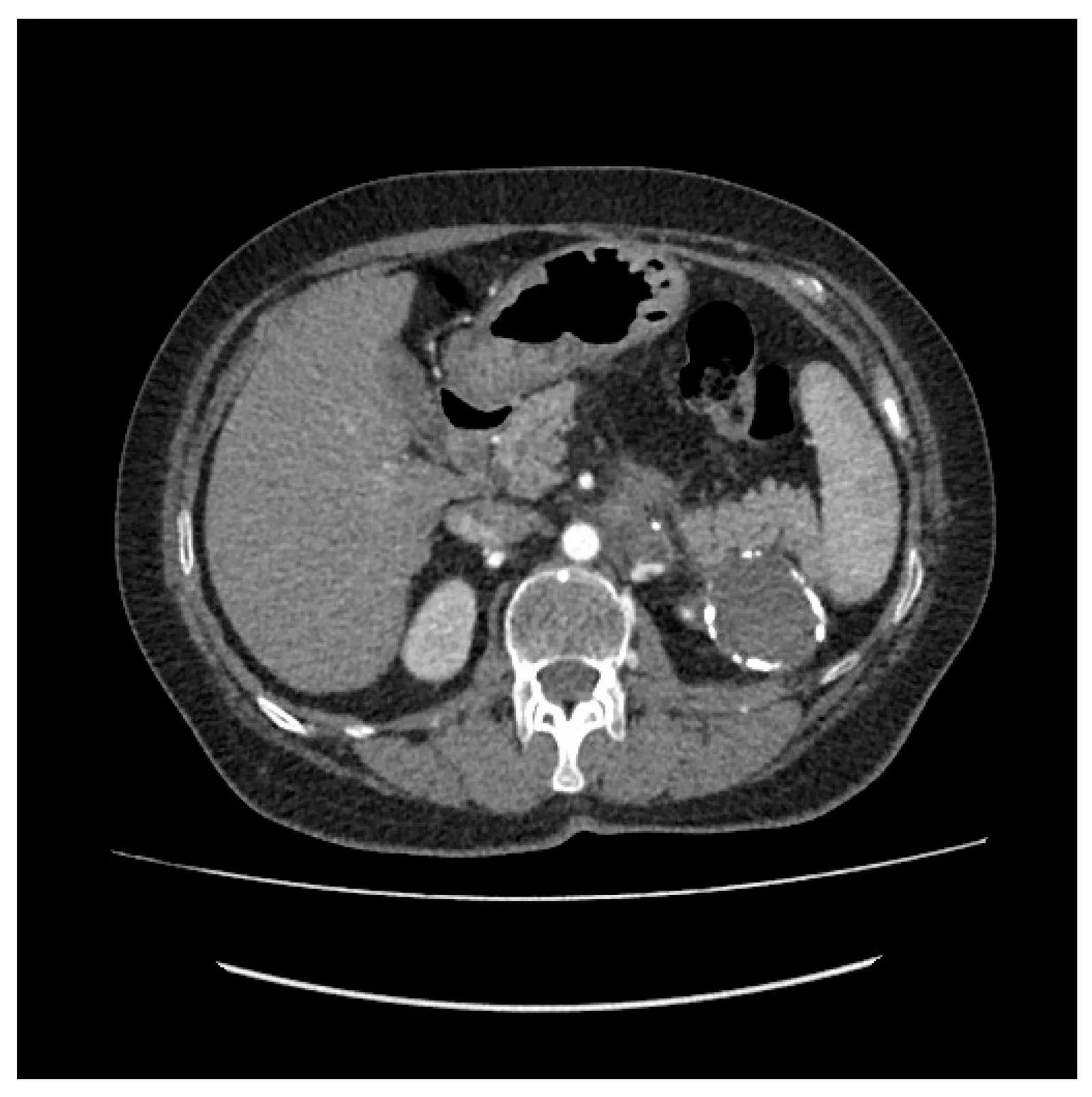
Postoperative follow-up CTA demonstrated no evidence of a fistula between the renal artery and renal vein.

Postoperative recovery was uneventful. During subsequent vascular follow-up, the patient remained asymptomatic, with no clinical evidence of recurrent arteriovenous shunting or complications related to the migrated pulmonary coil. Doppler ultrasonography and CTA confirmed complete occlusion of the renal vascular stump and exclusion of both the pseudoaneurysm and the arteriovenous fistula (Figure 5). Pulmonary perfusion remained preserved, with no evidence of pulmonary infarction or other complications attributable to the migrated embolization coil.

## 3. Discussion

The principal feature of this case is the combination of a delayed post-nephrectomy renal artery stump pseudoaneurysm and high-flow arteriovenous fistula, failed coil embolization with pulmonary migration, and successful laparoscopic vascular stump closure. The 30-year latency is unusual but not unprecedented: Gitlitz et al. reported presentation after 28 years and Kayser and Schäfer after 45 years [6,7]. The distinctive contributions of this report are therefore focused on the sequence of complications and rescue treatment rather than the latency alone.

The pathophysiology of delayed post-nephrectomy fistula formation is uncertain. Chronic changes in an arterial stump and historical en masse pedicle ligation have been proposed as possible contributing mechanisms [4,6,7]. These mechanisms cannot be confirmed in this patient. The nonspecific symptom of intermittent flank pain illustrates why a vascular lesion may not be suspected immediately decades after nephrectomy. High-flow arteriovenous communications can carry embolic devices into the draining vein and subsequently into the pulmonary circulation. Device stability depends on shunt calibre, velocity, landing zone, and deployment technique. Reports of transvenous rescue, balloon-assisted embolization, and combined arterial–venous approaches demonstrate possible preventive or salvage strategies [8,9,10,11]. In the present case, migration was documented and the fistula remained patent. The mechanism of migration is consistent with rapid flow through the fistulous communication, although the specific procedural contributing factors cannot be established from the available documentation. A covered stent, vascular plug, balloon-assisted embolization, or combined access may be considered in selected anatomies; their feasibility depends on proximal and distal sealing zones, branch anatomy, vessel diameter, and operator expertise [12]. A covered stent was not used because the direct high-flow communication was considered to carry a substantial risk of stent-graft displacement through the fistula into the renal vein, which could facilitate persistent arteriovenous flow rather than exclude the lesion. After failed embolization, laparoscopic stump closure interrupted the residual shunt without open reconstruction. The operation required exposure of the aorta and renal vascular pedicle, recognition of the markedly dilated left renal vein, and stapled closure of the stump. The migrated coil was located peripherally in the left pulmonary arterial circulation. Following an intraoperative medical consultation, retrieval was not attempted because the patient remained asymptomatic and haemodynamically stable and the anticipated procedural risk of retrieval was considered to outweigh its potential benefit. Conservative management of asymptomatic pulmonary coil migration has previously been reported [13]. Enoxaparin at 40 mg was administered until laparoscopic surgery. Postoperatively, enoxaparin thromboprophylaxis was continued for 30 days, and aspirin at 75 mg daily was prescribed for long-term treatment. During the two-year follow-up, two CTA examinations and Doppler ultrasonography confirmed complete exclusion of the renal artery stump pseudoaneurysm and arteriovenous fistula, with no pulmonary complications attributable to the migrated coil.

This single case supports the technical feasibility of laparoscopic closure after unsuccessful embolization in a carefully selected patient. It does not establish superiority over repeat endovascular treatment, open surgery, or robotic surgery. A robot-assisted approach was not considered in the present case because a robotic surgical system was not available at our institution at the time of treatment.

This case also illustrates the role of preoperative CTA-based anatomical assessment and contingency planning in selecting a minimally invasive vascular approach. Relatively long vascular stumps favoured safe dissection and stapling without prophylactic arterial cannulation. Prepared laparoscopic aortic clamps, a draped femoral access site, and available C-arm fluoroscopy provided options for rescue vascular control, although endovascular balloon occlusion would have required time to obtain arterial access. These preparations were intended to facilitate management of major haemorrhage and potential conversion to open surgery.

Selected comparable cases of delayed post-nephrectomy renal arteriovenous fistula are summarized in Table 1.

## 4. Conclusions

A high-flow renal arteriovenous fistula and renal artery stump pseudoaneurysm may present decades after nephrectomy. Coil embolization can be complicated by migration into the pulmonary circulation and persistent shunting. In this selected case, laparoscopic closure achieved anatomical exclusion after failed endovascular treatment; careful vascular planning and documented follow-up remain essential.

## Figures and Tables

**Table 1:** Selected comparable cases of delayed post-nephrectomy renal arteriovenous fistula.

Study	Interval from Nephrectomy (Years)	Symptoms	Treatment	Outcome
Gitlitz et al., 1963 [6]	28	Continuous right abdominal and flank bruit; no evidence of cardiac disease; chest and back pain with right-leg weakness	Surgical division and oversewing of the right renal artery supplying the fistula	Uneventful postoperative course; pain and neurological symptoms later recurred and remained unexplained
Kayser and Schäfer, 2013 [7]	45	Progressive dyspnoea and right flank pain; palpable thrill and systolic–diastolic bruit	Endovascular occlusion with Amplatzer vascular plugs	Complete occlusion; discharged the following day; asymptomatic recovery and stable plug position at 4 months
Present case	30	Episodic left flank pain without haematuria	Attempted coil embolization followed by laparoscopic ligation	Complete occlusion; asymptomatic; no pulmonary complications

## Data Availability

All data supporting this case report are included in the article.

## Abbreviations

The following abbreviations are used in this manuscript:

CT	Computed tomography
CTA	Computed tomography angiography
RAP	Renal artery pseudoaneurysm
RAVF	Renal arteriovenous fistula
